# Population Status of Vitamin B12 Values in the General Population and in Individuals with Type 2 Diabetes, in Southwestern Colombia

**DOI:** 10.3390/nu15102357

**Published:** 2023-05-18

**Authors:** Hernando Vargas-Uricoechea, Juan Patricio Nogueira, María V. Pinzón-Fernández, Valentina Agredo-Delgado, Hernando David Vargas-Sierra

**Affiliations:** 1 Metabolic Diseases Study Group, Department of Internal Medicine, Universidad del Cauca, Popayan 190003, Colombia; 2 Centro de Investigación en Endocrinología, Nutrición y Metabolismo (CIENM), Facultad de Ciencias de la Salud, Universidad Nacional de Formosa, Formosa 3600, Argentina; 3 Health Research Group, Department of Internal Medicine, Universidad del Cauca, Popayan 190003, Colombia; 4 Fellowship in Clinical Endocrinology and Metabolism, Universidad de Antioquia, Medellin 050010, Colombia; 5 Fellowship in Endocrinology, Diabetes and Metabolism, Universidad Pontificia Bolivariana, Medellin 050031, Colombia

**Keywords:** B12, vitamin, deficiency, diabetes, metformin

## Abstract

Vitamin B12 (B12) is necessary for the proper functioning of the central and peripheral nervous systems. Although there is no exact definition for B12 levels, a value of 200 pg/mL is compatible with deficiency, 200–299 pg/mL is considered borderline, and 300 pg/mL is considered normal. In population studies, the prevalence of B12 deficiency ranges between 2.9% and 35%. Furthermore, many medications, such as metformin [for type 2 diabetes mellitus (T2DM)], can cause B12 deficiency. The objectives of this study were to determine the population status of B12 in southwestern Colombia (and the status of B12 in subjects with T2DM). In the total population (participants with and without T2DM), the prevalence of B12 deficiency was 17.8%; that of borderline was 19.3%; and that of normal levels was 62.9%. The prevalence of deficiency increased with age and was significantly higher in those aged ≥60 years (*p* = 0.000). In T2DM subjects, the prevalence of deficiency was significantly higher concerning those without T2DM (*p* = 0.002) and was significantly higher in those who received >1 gm/day of metformin (*p* = 0.001). Thus, the prevalence of deficiency and borderline levels of B12 in our population was high, particularly in those >60 years of age. B12 deficiency was significantly higher in individuals with T2DM than in individuals without T2DM, especially among those receiving high doses of metformin.

## 1. Introduction

Vitamin B12 (B12) or cobalamin is part of a set of structures called corrinoids with biological activity in humans; B12 is found naturally in animal foods, including fish, meat, poultry, eggs, milk, and milk products [1]. 

The main factor associated with the development of B12 deficiency in high-income countries is of autoimmune origin (pernicious anemia); however, in low-income countries, the main associated factors are low intake of foods rich in B12 (especially those of animal origin), although other factors have been described, such as gastrointestinal infections and intestinal parasitism, among others [1,2].

B12 is recognized as a cofactor, and has a fundamental role in DNA-related methylation processes; therefore, B12 deficiency can induce DNA disruption. Additionally, B12 is necessary for the methylation of myelin, neurotransmitters, and membrane phospholipids and is essential for the integrity of the central and peripheral nervous systems [1,2,3].

The recommended intakes of B12 have been established to maintain the levels of the vitamin within a normal range (in the blood), based on the fact that about 50% of the B12 coming from the diet is absorbed. In the United States (US), for instance, an intake of 2.4 µg/day is recommended for adults. Higher intakes are recommended for vulnerable groups (children, pregnant individuals, and lactating individuals) [4,5,6].

Meanwhile, in the United Kingdom, the recommended intake is 1.5 µg/day (and 4 µg/day as adequate intake); this recommendation would cover the requirements or needs of the adult population in Europe [6,7,8].

However, despite the established recommendations, there is no universally accepted value to define the “deficiency” or “sufficiency” of B12. Nevertheless, a serum value < 200 pg/mL is compatible with deficiency, a value of 200–299 pg/mL is considered borderline, and a value ≥ 300 pg/mL is considered normal. These cut-off points to define B12 values in the population are accepted by several international organizations (including the World Health Organization) and have been widely used in other studies [8,9,10,11].

Based on these thresholds, B12 deficiency is widely variable, being more frequent in the elderly, women of childbearing age, children, and adolescents. In the US National Health and Nutrition Examination Survey (NHANES) between 1999 and 2004, the prevalence of B12 deficiency was 2.9%, 10.6%, and 25.7%, according to the cut-off points of <200, <271, and <346 pg/mL, respectively, being higher in older adults and women [10,11].

In Latin America, the population prevalence of B12 deficiency is between 2.5% and 35%, and borderline levels can reach up to 60% [11,12,13].

B12 deficiency can be exacerbated by the use of some medications (nitrous oxide, proton pump inhibitors, H2 receptor antagonists, inter alia); however, the use of metformin in individuals with type 2 diabetes mellitus (T2DM) is better documented. In fact, it has been reported that, on average, 6 to 30% of patients receiving metformin are deficient in B12, and it has been found that B12 levels are inversely related to the duration and use of metformin [14,15].

This study aimed to determine the prevalence of deficiency, borderline, and normal levels of B12 in a population from southwestern Colombia; additionally, we also evaluated the status of B12 in subjects with T2DM.

## 2. Methods

### 2.1. Participants and Setting

This population-based, cross-sectional, prospective study was approved by the research ethics committee of the Universidad del Cauca-Colombia (ID: 4656, January 2018). All procedures were conducted in compliance with the Helsinki declaration for research on human beings.

The study population consisted of ambulatory individuals from the urban area of Popayán-Colombia, who were invited to participate in the study through active visits to the different neighborhoods and communes of the city, between 5 June 2018 and 15 January 2022. In 2018, Popayán had an estimated population of 270,000 inhabitants [16].

A total of 5679 adults (≥18 years old) signed the informed consent after an explanation of the purposes and objectives of the study was given; they then filled out a questionnaire about previous health history. Subsequently, a measurement of folic acid in the blood was performed; a normal value of folic acid was defined when it was between 3 and 17 ng/mL [17,18,19].

**Figure 1:**
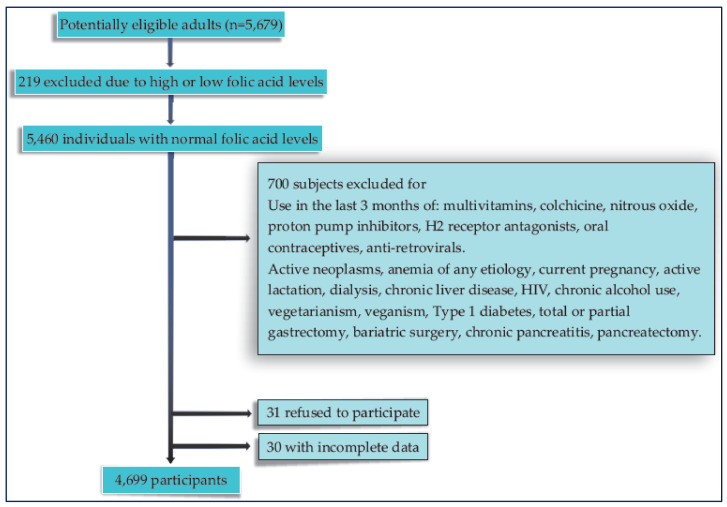
Flow chart of the participants included in the study.

Those participants with low or high folic acid values were excluded, as well as those with an active intake of medications or health conditions that could potentially alter the B12 metabolism; finally, 4699 individuals were included in the study (Figure 1). Additionally, subjects who had a diagnosis of T2DM (considering the history confirmed by the treating physician) and who met the inclusion criteria were also included in the analysis.

### 2.2. Measurement of B12 Values

The blood samples were taken via venipuncture, under fasting conditions; then, they were centrifuged, frozen at −20 °C, and stored. The B12 tests were performed in the serum using a chemiluminescent immunoassay (IMMULITE^®^ 2000 Systems Analyzers; Siemens, Munich, Germany), and the coefficient of variation intra-assay and inter-assay was 7.0% and 6.0%, respectively. The results were classified as follows: <200 pg/mL (deficiency); 200–299 pg/mL (borderline); and ≥300 pg/mL (normal) [8,9,10,11].

### 2.3. Statistical Analysis

The information was collected in an Excel database and analyzed with the statistical software IBM SPSS version 25.0. In the primary analysis, the population’s B12 status (deficiency, borderline, and normal levels) was described based on global and age-specific prevalence and 95% confidence intervals (95% CI). The relative inequality and its 95% CI were used as a measure of association strength. The chi-square test (*X*^2^) was applied for linear trends. The variables on a numerical scale were analyzed based on frequency distributions, measures of central tendency, and variability (mean, median, and interquartile range (IQR)).

The median and interquartile range for B12 were established, since the data distribution did not meet the statistical normality criterion (Kolmogorov–Smirnov test). To analyze the relationship between the population status of B12 (in individuals with or without DM2) with or without the use of metformin and according to the dose used (<1 gm/day or ≥1 gm/day), we used the differences in proportions, the measure of the strength of association, 95% CIs, *X*^2^ from the Mantel and Haenszel test, and *X*^2^ for linear trends. In all cases, a level of statistical significance (α ≤ 0.05) was established [18].

## 3. Results

### 3.1. Baseline Characteristics of The Subjects

Most participants were men, and the median age was similar in both sexes; however, the mean age was significantly higher in participants with T2DM. The average body mass index (BMI) classified the population as overweight, and the vast majority of T2DM subjects were receiving metformin. Among these, the number of participants receiving parenteral or oral management did not differ according to the metformin dose (Table 1).

### 3.2. Status of B12 in The Total Population

**Figure 2:**
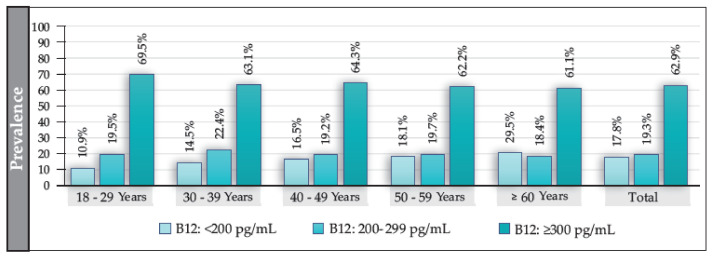
Prevalence of deficiency, borderline, and normal levels of B12 in the total population and according to age group (in years).

The median B12 value for the total population (participants with and without T2DM) was 341 pg/mL (IQR: 238–514); in women, it was 347 pg/mL (IQR: 229–509), and in men, it was 351 pg/mL (IQR: 221–501) (*p* = 0.118). The prevalence of B12 deficiency in the total population was 17.8%; that of borderline levels was 19.3%; and that of normal levels was 62.9% (Figure 2). The prevalence of B12 deficiency exhibited an increasing and significant trend according to age (*p* = 0.000) and was independent of sex. Additionally, B12 deficiency was significantly higher in ≥60 vs. <60 years (OR: 1.4 (95% CI: 1.2–1.6; *p* = 0.000)). No significant differences were found in the prevalence of borderline values either by sex or by age, except in participants aged ≥60 years, where the prevalence was significantly higher in men (22.7%) than in women (16.6%) (OR: 1.4 (95% CI: 1.1–1.7; *p* = 0.005)). We also found no differences in the levels of B12 concerning BMI or in the presence of smoking.

### 3.3. Status of B12 in The Population without T2DM

**Figure 3:**
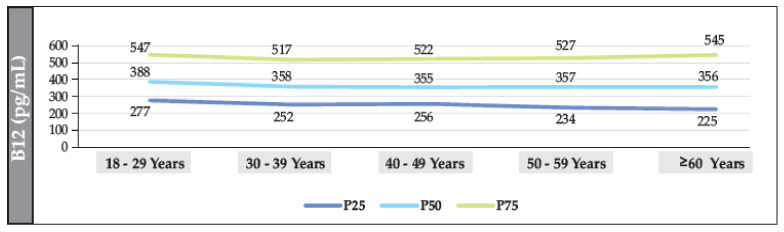
Median distribution (IQR) of B12 (according to age range) in the total population of individuals without T2DM.

**Figure 4:**
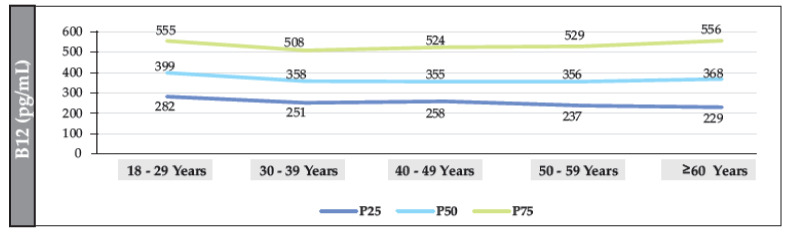
Distribution of the median (IQR) of B12 (according to age range) in the population of individuals without T2DM (women).

**Figure 5:**
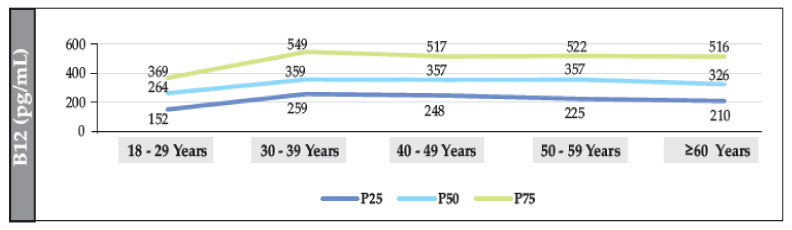
Distribution of the median (IQR) of B12 (according to age range) in the population of individuals without T2DM (men).

The median B12 value in participants without T2DM was 361.2 pg/mL (IQR: 222–548). The median was also found to have a linear trend that decreased significantly with age (18–29 years vs. ≥60 years (*p* = 0.002)). The median value in women and men was 354 pg/mL (IQR: 243–541) and 349 pg/mL (IQR: 236–549), respectively (*p* = 0.098). The prevalence of B12 deficiency was 12.7% (95% CI: 11.7–13.8%), and the prevalence of borderline levels was 19.2% (95% CI: 18.1–20.4%) (Figure 3, Figure 4 and Figure 5).

### 3.4. Status of B12 in the T2DM Population

The median B12 value in the T2DM population was 187.8 pg/mL (IQR: 144–289.9), and 187 pg/mL (IQR: 148–290) and 190.5 pg/mL (IQR: 138.4–289) in women and men, respectively.

Similarly, the median B12 value according to age was as follows: 18–29 years: 270 pg/mL (IQR: 184.7–390.5); 30–39 years: 192 pg/mL (IQR: 133–265.5); 40–49 years: 186.4 pg/mL (IQR: 150–320.2); 50–59 years: 190 pg/mL (IQR: 120–272); and ≥60 years: 175.3 pg/mL (IQR: 142–274).

No differences were found in the median B12 value according to sex (*p* = 0.21), age ranges (*p* = 0.09) or between participants with or without T2DM (0.07).

The prevalence of T2DM in the total population was 11.6%, being significantly higher in men. More than two-thirds of these participants were aged ≥60 years, and the prevalence of B12 deficiency was significantly higher in subjects with T2DM than in those without T2DM.

There were no significant differences in borderline B12 values between participants with T2DM and those without T2DM.

In total, 96% of the participants with T2DM received management with metformin and, among them, the prevalence of B12 deficiency was found to be significantly higher in those who received >1 gm/day (Table 2).

No differences were found according to sex (*p* = 0.252) or age range (*p* = 0.289), and the prevalence of deficiency, borderline, or normal levels of B12 was independent of the use of parenteral and other oral medications (*p* = 0.282 and *p* = 0.418, respectively).

## 4. Discussion

The population status of B12 is variable; for instance, the geometric mean of the B12 serum concentrations in the US population (NHANES, 2003–2006) was 500 pg/mL (95% CI: 489–511), without finding marked differences according to age and sex. The prevalence of deficiency in the total population was 2.0%, 1.5%, 2.6%, and 3.9% in subjects aged 20–39, 40–59, and ≥60 years, respectively [10,11,17].

This is in contrast with our results, where we found a much higher prevalence of deficiency and borderline levels than in the NHANES study (although in our population, the prevalence of deficiency and borderline levels was also higher in those ≥60 years of age). However, in other Latin American countries, the prevalence of deficiency is stipulated as being 9–68% [12,13].

The differences in prevalence can be explained in several ways. For example, some countries in this geographic area have folic acid fortification programs, and an inverse relationship between high levels of folic acid and B12 deficiency has been described [12,13].

Nevertheless, in our study, only individuals with normal folic acid values were evaluated, which makes it possible to exclude (at least in part) a specific influence of folic acid levels on the results, although a residual confounding phenomenon cannot be completely ruled out.

Thus, the high prevalence of deficiency and borderline levels of B12 in our study can also be explained by other factors, such as nutritional factors, B12 malabsorption or intrinsic factor (IF) deficiency, or due to autoimmune and non-autoimmune chronic atrophic gastritis, *H. pylori* infection, or the state of the intestinal microbiome. Hereditary disorders or specific polymorphisms that can affect the B12 transporter proteins are also potential factors [1,2,20].

In this sense, in the “National Survey of the Nutritional Situation, ENSIN-Colombia, 2015” it was found that, in women of childbearing age, the prevalence of B12 deficiency was 3.5%, and that of “risk of deficiency” (200–299 pg/mL) was 14.7%. In pregnant women, the prevalence of deficiency was 11.6% and the “risk of deficiency” was 33.9% [20,21].

This suggests that, in Colombia, the prevalence of at least one definition of “risk” for B12 deficiency is high, and that the B12 population status is also highly variable.

Taking into account that B12 is found naturally in foods of animal origin (fish, meat, poultry, eggs, and dairy products), but also in fortified cereals, in Colombia (in adults 18–64 years), the prevalence of the consumption of different types of meat or eggs is 94.3% and 95.5%, that of dairy is >80%, and that of cereals such as rice or pasta, bread, and corn arepa is 99.2%, 88.2%, and 85.3%, respectively [21,22].

Therefore, the intake of foods with a high content of B12 in Colombia is highly prevalent, and probably does not further explain the prevalence of B12 deficiency or borderline levels found in our population.

On the other hand, in Colombia, the prevalence rate of *H. pylori* infection is around 70%. These data go in the same direction with the high rate of multifocal atrophic chronic gastritis found in the geographic area where this study was conducted [23,24].

In this sense, the absorption of B12 requires gastric acid to be able to free itself from different protein complexes in food and to be able to bind to IF, which is produced in the gastric body and is essential for the absorption of B12. Although pernicious anemia does not seem to be related to *H. pylori*, it is suggested that it may be initially triggered by this infection, with severe inflammation and atrophy of the gastric body and the consequent loss of IF secretion (and B12 absorption). Indeed, *H. pylori* eradication may improve B12 absorption and levels in individuals with atrophic gastritis [25,26,27].

Unfortunately, in Colombia, there is no population study with an adequate sample size that allows knowing the true frequency of autoimmune atrophic gastritis (AAG). In this study, we also did not evaluate the autoimmune component that is related to AAG.

Therefore, it can be argued that the high prevalence of both *H. pylori* infection (and multifocal atrophic chronic gastritis) in our setting explains (at least in part) the high prevalence of B12 deficiency and borderline levels.

In the same way, the fact that intestinal parasitism has a role in the absorption of multiple vitamins (including B12) cannot be ignored; for instance, *Giardia duodenalis* is known to cause malabsorption, with preliminary studies finding improvements in B12 serum levels after treatment for giardiasis. It has even been suggested that *Giardia duodenalis* is associated with less microbiome diversity and a higher abundance of Prevotella (associated with the diminished presence of cobalamin synthesis genes). Additionally, *Giardia duodenalis* can colonize the proximal small intestine, where B12 is absorbed; therefore, giardiasis can lead to B12 deficiency [28,29].

If one takes into account that the general prevalence of parasitism in Colombia is 65.9%, it is probable that the high rate of intestinal parasitism in Colombia may affect the population’s B12 status [30].

Other factors (such as hereditary factors or specific polymorphisms) have not been evaluated on a large scale in our environment, and the real population distribution of these factors is unknown.

Finally, in the population with T2DM, the prevalence of B12 deficiency was high, and almost all subjects were receiving metformin; among them, doses ≥1 gm/day were associated with a higher prevalence of deficiency. These data support the findings of other, similar studies, where the percentage of the reduction in B12 levels attributable to metformin use ranged from 17.8% to 26.8% in cross-sectional studies and from 6.3% to 18.7% in clinical trials with 6–16-week durations [31,32,33].

Several mechanisms may explain the relationship between metformin intake and B12 deficiency. For example, metformin may alter intestinal motility, which could cause bacterial overgrowth in the small intestine and the inhibition of IF/B12 complex absorption in the ileum distal; another mechanism proposes that metformin antagonizes the calcium and interferes with the calcium-dependent IF–B12 complex binding to the ileal cubilin receiver; in fact, the reversal of metformin-associated B12 malabsorption by calcium supplementation greatly supports this mechanism. Additionally, metformin can positively charge the cell membrane surface in the ileum, potentially displacing calcium through different repulsive forces. This displacement alters the mechanism of action of the said complex, leading to a state of B12 malabsorption. Finally, since bile is secreted in the duodenum, the above theory adds that metformin may inhibit bile-mediated B12 uptake in the distal ileum, impairing the enterohepatic circulation of B12 [33,34,35,36].

Otherwise, although we did not have complete information regarding the concentration and dose of other parenteral and/or oral treatments for T2DM in our study, we did not identify any differences between the frequency of use of said drugs in subjects receiving metformin. 

We also found that the prevalence of B12 deficiency in participants receiving metformin at doses >1 gm/day was higher (relative to those receiving lower doses), especially in men. It is difficult to explain this result; however, it must be taken into account that, in this study, we did not evaluate the time of metformin use or the average dose received by the participants, and in this regard, it is clear that there may be related confounding factors; for example, B12 deficiency may be higher in individuals receiving doses of metformin close to 2 gm/day or also in individuals with prolonged use of the medication (for example, for a period of time greater than 5 years) and these findings may be more frequent in men (which can potentially explain the difference found compared to women).

Finally, the differences found between those who received metformin ≥1 gm/day and those receiving <1 gm/day suggest that a dose >1 gm/day could be considered a powerful B12 deficiency indicator in T2DM [37,38,39].

Our study has some weaknesses; for example, we only used serum levels as a biomarker of B12 status, and a limitation of this biomarker is that it assesses total circulating B12, of which near to 80% is bound to haptocorrin, and therefore is not bioavailable for cellular uptake. We also did not measure the holo-transcobalamin or methylmalonic acid levels; these two markers are very useful to assess the status of B12 [1,2,9].

We also did not evaluate the IF values, so we cannot establish how many of the subjects with B12 deficiency could have pernicious anemia as the underlying cause. We also did not determine the history of chronic gastritis with or without *H. pylori* infection, either via endoscopy (with histopathology) or the breath test. In fact, the chronic atrophic gastritis may explain (at least in part) the high prevalence of B12 deficiency in people ≥60 years of age, as we found in the study.

Additionally, the design characteristic of our study (cross-sectional) allows us to establish the population’s B12 status at a specific moment in time, without necessarily reflecting its dynamic status, which can change over time or due to various nutritional and environmental circumstances, among others.

On the other hand, some strengths can be highlighted in this study. For instance, the inclusion of individuals with normal blood levels of folic acid made it possible to analyze the data without any potential confounding effects caused by its excess or deficiency. We also excluded individuals with health conditions and medication intake that could eventually modify B12 levels. We were also able to assess a significant number of participants with T2DM, allowing us to analyze the distribution and status of B12 in this population.

The results of this study should only be extrapolated to similar populations; therefore, the findings described may not necessarily reflect the B12 status of specific populations or those with concomitant health conditions (those pregnant or lactating, children, or patients with active cancer, advanced kidney disease, HIV, and liver disease, among others).

## 5. Conclusions

In southwestern Colombia, more than a third of the population presented deficiency or borderline levels of B12. The results suggest that the main factors that may explain this frequency are, among others, the high rate of multifocal atrophic chronic gastritis, added to the high frequency of *H. pylori* infection. Similarly, the high prevalence of B12 deficiency in T2DM is probably due to the use of high doses of metformin. 

## Figures and Tables

**Table 1:** Baseline characteristics of the participants (total population, without T2DM and with T2DM).

Characteristics	Total Population (*n* = 4699)	Population without T2DM (*n* = 4153)	Population with T2DM (*n* = 546)	*p*-Value
Age in years (median, IQR)	49.2 (35–60)	48.1 (36–62)	59.7 (43–71)	0.048 **
Male number (%)	2631 (56)	2284 (55)	349 (64)	0.428
BMI, kg/m^2^ (mean ± SD	26.4 ± 4.8	26 ± 3.3	27 ± 4.1	0.281
Smoking current –yes-, number (%)	455 (9.7)	365 (8.8)	57 (10.4)	0.371
Serum folate (ng/mL), median (IQR)	10.8 (5.2–13.7)	9.9 (4.3–12.8)	8.7 (4.1–11.4)	0.392
Number (%) of participants receiving metformin	-	-	524 (96)	
Number (%) of participants receiving metformin at doses <1 gm/day *	-	-	256 (49)	0.182
Number (%) of participants receiving metformin at doses ≥1 gm/day *	-	-	268 (51)	
Duration of metformin use (years), mean ± SD	-	-	5.2 ± 2.1	
Use of parenteral medications (GLP-1RA and/or insulin in participants receiving metformin at doses <1 gm/day (number of participants, %)			24 (9.4)	0.096
Use of parenteral medications (GLP-1RA and/or insulin in participants receiving metformin at doses ≥1 gm/day (number of participants, %)			30 (11.2)	
Use of other oral medications in participants receiving metformin at doses <1 gm/day (number of participants, %)			63 (24.6)	0.099
Use of other oral medications in participants receiving metformin at doses ≥1 gm/day (number of participants, %)			70 (26.2)	

* The daily dose of metformin was calculated as the mean dose taken over the last 6 months; ** Population with T2DM vs. population without T2DM. Abbreviations: BMI: body mass index, IQR: interquartile range, SD: standard deviation, T2DM: type 2 diabetes mellitus.

**Table 2:** Prevalence of B12 values in participants with and without T2DM, and according to the dose of metformin received.

Characteristics	Total Population, Number (%)	Population without T2DM	Population with T2DM	
			Male, Number (%)	Women, Number (%)
Prevalence of T2DM	546 (11.61)	-	349 (64)	197 (36)
		-	OR: 1.2 (95% CI: 0.99–1.4); *p* = 0.083	
Prevalence of T2DM in ≥60 years old, *n* = 426 (78.1%) (*p*-value)	-	-	277 (65)	149 (35)
		-	0.01	
Prevalence of B12 deficiency [% (95% CI]	-	12.7% (95% CI: 11.7–13.8%),	Male and women: 56.8% (95% CI: 52.5–61.0%)	
	-	OR = 9.1 (95% CI: 7.5–10.9); *p* = 0–002		
Prevalence of B12 borderline values	-	19.2%	Male and women: 20.7%	
	-	*p* = 0.132		
Participants with T2DM who received management with metformin, number (%)	-	-	524 (96)	
Prevalence of B12 deficiency in participants receiving metformin management (≥1 gm/day) vs. (<1 gm(day)			Male and women: 72.8% vs. 21.2% (*p* = 0.001)	

Abbreviations: CI: confidence interval, OR: odds ratio, T2DM: type 2 diabetes mellitus.

## Data Availability

The data presented in this study are available on request from the corresponding author.
